# Responses of Descending Visually-Sensitive Neurons in the Hawkmoth, *Manduca sexta*, to Three-Dimensional Flower-Like Stimuli.

**DOI:** 10.1673/031.009.0701

**Published:** 2009-03-17

**Authors:** Jordanna D. H. Sprayberry

**Affiliations:** ^1^ Current Address: University of Arizona, ARL Division of Neurobiology, Gould-Simpson Bldg. Room 611, Tucson AZ 85721; University of Washington Box 351800, Department of Biology, Seattle WA 98155-1800

**Keywords:** flower tracking, cervical connectives, object-detection

## Abstract

Hawkmoths rely on vision to track moving flowers during hovering-feeding bouts. Visually guided flight behaviors require a sensorimotor transformation, where motion information processed by the optic ganglia ultimately modifies motor axon activity. While a great deal is known about motion processing in the optic lobes of insects, there has been far less exploration into the visual information available to flight motor axons. Visual information recorded at this stage has likely arisen from multiple visual pathways, and has potentially been modified by outside sensory information. As a first step, understanding the sensorimotor transformation from transduction of moving flower signals to active flower tracking behavior requires that the visual information available to the thoracic flight control centers be assayed. This paper investigated the response of descending visually sensitive neurons in the cervical connectives of the hawkmoth, *Manduca sexta* L. (Lepidoptera: Sphingidae), to flower-like stimuli. Because flower structure lends itself to oscillatory (vibratory) motion, the stimuli used in these experiments were discs oscillating in each axis of motion (horizontal, vertical, and looming). Object-sensitive descending-neurons (OSDNs) respond to multiple directions of object motion and do not clearly sort into classes of directional tuning. The broad spatial distribution of directional sensitivities exhibited by OSDNs indicates that the direction of object motion may be encoded on a population scale. Although OSDNs exhibit broad frequency response curves, over the range of frequencies that *M. sexta* are able to track (0–2 Hz) OSDNs exhibit monotonically increasing response. Additionally, OSDNs respond to discs oscillating at frequencies as high at 6 Hz, indicating that the visual information being sent to thoracic motor control centers is not likely the limiting factor in flower tracking ability.

## Introduction

How visual information is processed to affect coordinated motor patterns remains a central question in insect flight control. While significant inroads have been made in understanding processing of visual information in the optic ganglia, less attention has been given to how these highly derived visual signals are modulated to communicate with flight control centers. Developing an understanding of how visuomotor signals correlate with output behavior requires a synergy between behavioral and neurop-hysiological investigations. Flower tracking behavior in hawkmoths is an ideal system for such a synergistic approach.

Hawkmoths are hovering feeders that exhibit a compensatory tracking behavior when challenged with flower motions (Farina et al. 1994; Moreno et al. 2000; Sprayberry and Daniel 2007). The need to track flowers is indeed a consistent challenge for hawkmoths; in addition to environmental winds, the air currents from a hawkmoth's wing-beats are capable of inducing flower motion (Sane and Jacobson 2006, http://faculty.washington.edu/danielt/sprayberry06.mov). This behavior is visually guided (Farina et al. 1994) and therefore likely to be driven by descending visual inputs (Kern and Varju 1998; Kern 1998). To begin to understand what key visual information is required to drive flower tracking, it is necessary to compare the visual information being sent to thoracic flight control centers with the output flower tracking behavior. In a companion behavior study, we examined the ability of *Manama sexta* L. (Lepidoptera: Sphingidae) to track flowers that were moving in different directions (horizontal, vertical and looming) and at varying frequencies (0–3 Hz) (Sprayberry and Daniel 2007). *M. sexta* are best able to track flowers moving at a frequency of 1 Hz, and by 3 Hz their ability to track is compromised. Additionally, *M. sexta* are better at tracking flowers moving horizontally and vertically than they are at tracking flowers moving in the looming axis (towards and away from the moth). Flower tracking behavior could be constrained by the ability of the visual system to process moving flower signals, the aerodynamic capability of the moth to physically follow moving flowers, or a combination of both.

This study endeavored to assess what information about direction and frequency of moving flowers is made available to the thoracic motor system. Additionally, these data are used to hypothesize whether or not flower tracking ability in *M. sexta* is limited by the visual system. The cervical connectives were an ideal recording location to conduct this investigation for several reasons. First, visually-sensitive neurons at this level are downstream of primary visual-processing and higher-order integration centers. Responses of these neurons will likely integrate any necessary outside sensory information. Finally, these neurons terminate in the thoracic ganglia (Rind 1983a), where they could potentially synapse with steering muscle motor axons (Rind 1983b). Here the responses of visually-sensitive descending neurons to flowerlike object motion is examined in all three motion axes (horizontal, vertical, looming) and at varying frequencies (0–6 Hz).

## Materials and Methods

### Animals and preparation

**Figure 1.**
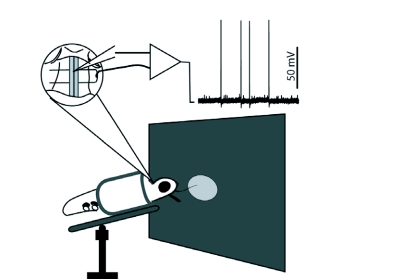
A lateral view of the electrophysiology setup. The zoom insert shows the cervical connectives, between the head and thorax, supported by a tungsten wire bent into a U. The tungsten support also served as a ground. Experiments used pulled quartz glass electrodes, with an approximate tip diameter of 50 nm, permitting single unit recordings. *Manduca sexta* were restrained in a tube and placed ventral side up in front of a rear projection screen.

The adult *M. sexta* used in these experiments were reared at the University of Washington. Moths were cold anesthetized prior to dissection. Animals had their legs removed and were immobilized in a plastic restraining tube. They were then fixed in place by waxing the thorax to the restraining tube. The head was tilted backwards and waxed to the prothorax. The soft tissue in between the prothorax and the head was removed to expose the cervical connectives. A small U-shaped tungsten rod placed under the nerve cord served as a physical support (Figure 1), a local ground for recordings using an extracellular differential AC amplifier, and the general ground for recordings made with an intracellular amplifier (see below). A thin silver wire was placed in the ventral-posterior section of the left eye to act as a general ground for recordings using an extracellular amplifier. The antennae and proboscis were left intact during experiments. Moths had a minimum of 35 minutes to recover from cold anesthesia before experiments began. Moths were placed in front of a fixed rear-projection screen ventral side up, allowing single-unit recording from the descending nerve cord. This orientation has commonly been used for recordings from descending cells in insects (Burrows and Fraser Rowell 1973; Olberg 1983; Kanzaki et al. 1991;; Frye 1995; Kern and Varju 1998).

### Electrophysiological recordings

Individual cervical neurons were recorded using pulled quartz glass electrodes (P2000, Sutter) filled with 0.1M KCl solution, which had an approximate tip diameter of 50nm and a resistance of 130MΩ (as measured in saline). Electrodes were stepped through the cervical connectives using a World Precision Instruments DC 19 micromanipulator that permitted step sizes of 0.5–10nm (www.wpiinc.com). Because cells were not neuroanatomically reconstructed and identified, this manuscript refers to all cells recorded from as ‘units’. Initial experiments used a model 1800 AM Systems (www.a-msystems.com) 2channel extracellular-amplifier (units AJ). Later experiments used a model 5 Getting (www.gettinginstruments.com) intracellular-amplifier (units KP). Neural signals were digitally recorded (Powerlab 8sp, Chart 4.1) at a rate of 10KHz, with the exception of Unit A (recorded at 4 KHz).

The recording protocol involved a ‘seek and test’ method, stepping an electrode through the cervical connective while listening for any spiking neurons. To increase the likelihood of finding a visually sensitive cell a looping left-right wide-field sinusoidal grating was shown on the projection screen, which is often excitatory to descending visual-neurons (Mark Willis, personal comm.). When any spiking neuron was encountered, it was tested for sensitivity to object motion in all three motion axes (horizontal, vertical and looming). A narrow class of visually-sensitive neurons would not have been detected: those that had no baseline spiking activity, did not spike when impaled by the electrode, and were not excited by a left-right sinusoidal grating.

### Visual stimuli

Computer-generated visual stimuli were displayed using a Boxlight (www.boxlight.com) DLP projector (model XD10m). The projector was placed behind the screen depicted in Figure 1, so that animals viewed rear projection stimuli. The projector had a luminance refresh rate of 360Hz, but total luminance levels never fell below 50%. However, each individual color channel (red, green, and blue) had a flicker rate of 120Hz, with that color's luminance falling completely to zero before the next cycle. Electroretinograms were recorded from *M. sexta* to determine if they have a flicker fusion rate higher than 120Hz, and could therefore resolve the flicker of individual color channels (see above).

For the first ten units (A-J), visual stimuli were generated using MatLab Psychophysics Toolbox software (www.psychotoolbox.org) (Brainard 1997; Pelli 1997). Objects (discs) were animated with a saw-tooth position profile, resulting in a square-wave velocity pattern. The order of stimuli was not randomized. For ease of programming and increased flexibility, visual stimuli were switched to an openGL based motion animation system: Vision Egg (www.visionegg.org, Straw et al. 2006). The last seven units (K-P) were subjected to Vision Egg stimuli. These stimuli were identical to Psychophysics Toolbox stimuli with two exceptions. First, object animation used a sinusoidal position profile, resulting in a cosinusoidal velocity pattern; which more closely resembled the motion of natural flower vibrations than a square wave. Second, only the first trial was presented in order. Subsequent trials were randomized (one of seven units, unit P, showed an order effect of preceding direction of motion, ANOVA, *p* < 0.05). Data from both types of stimuli were pooled, as there were no statistical effects of MatLab Psychophysics stimulation versus Vision Egg stimulation on either response to individual directions of motion, or the specificity of units’ responses to different directions of motion (Students T Test, ANOVA).

Animals were placed in the recording rig 5 cm from the screen (Figure 1). All stimuli were light discs (51 cd/m^2^) against dark backgrounds (1.9 cd/m^2^) unless otherwise stated. All luminance measurements of stimuli were taken with a Gossen Mavolux 5032C light meter (www.gossenphoto.de). The light discs were always 5 cm in diameter, except when simulating looming axis motion by changing disc size. The disc subtended an angle of 45 degrees on the moth's retina. At the beginning of each experiment, a stimulus disc was centered in the animal's frontal field of view. All motion animation of the disc during that experiment began and ended at that location.

Although recordings necessarily place animals in an unnatural position, these experiments were designed to resemble natural stimuli as closely as possible. Because natural flowers are physically incapable of unidirectional movement, motion presentations within a given axis were an oscillating disc. This disc was 5 cm in diameter which is not only within the size range of flowers *M. sexta* pollinate, but also matched the size of the artificial flower used in a companion behavior study (Sprayberry and Daniel 2007). While flower tracking may be visually guided, feeding behavior in hawkmoths integrates both olfactory and visual cues (Raguso and Willis 2002, 2005). If olfactory information modifies encoding of visual information about moving flowers, it has likely been integrated into responses of the descending neurons recorded from in these experiments. In an attempt to place the nervous system in a relevant sensory context, linden honeysuckle flower-odor (produced by L'Occitane en Provence, www.loccitane.com) was released near the moths' antennae during recordings. Moths were exposed to floral odor for at least 5 minutes before recordings commenced, and odor delivery remained constant throughout the experiments. The effects of changing odor conditions were not tested.

### Electroretinogram recordings

Electroretinograms (ERGs) were recorded from *M. sexta* to test for flickering artifacts from the projector used in these experiments. These recordings used the same type of pulled glass electrode and intracellular amplifier as the cervical neuron recordings (see above). While the projector's overall luminance flickered from 100 to 50 percent at a rate of 360 Hz, the individual color channel luminance flickered from 100 to 0 percent at a rate of 120Hz. As motion-detection pathways in insects have been shown to be broadly green sensitive (McCann and Arnett 1972), and feeding in *M. sexta* appears to be mediated by blue receptors (Cutler et al. 1995), visual processing of object stimuli might be affected by this 120 Hz flicker. If individual photoreceptors are able to resolve a 120Hz flicker at the luminance levels used in these experiments, ERGs should show multiple responses to a single flash of a projected disc. The projector updated information at 60 Hz, and the flashing stimuli were constructed based on this frame rate. A projected light disc was flashed for a twoframe duration (33.33 ms) with an inter-flash interval of 30 frames (500 ms). Because of the projector's luminance flicker, each 2-frame flash actually consisted of 6 on-off flickers in each color channel (r-g-b-r-g-b-r-g-b-r-g-b-r-g-b-r-g-b).

### Direction test

The direction test consisted of three sequential 1 second long presentations of an animated disc (see above) representing flower motion in three orthogonal axes: (1) a 5 cm disc was oscillated in the horizontal plane (left-right) at a frequency of 2 Hz and amplitude of 0.5 cm; (2) the same image was oscillated in the vertical (up-down) plane at a frequency of 2 Hz and amplitude of 0.5 cm; (3) motion in the looming axis (approach-recede) was simulated by using an expanding and contracting disc as a 2D projection of a flower oscillating at 2 Hz frequency and 0.5 cm amplitude (based upon a 5 cm distance to the flower). In each case, the disc appeared on the screen and remained stationary for 1 second before and after any motion animation. The disc also remained stationary for a 1 second inter-stimulus interval between oscillations.

### Luminance test

Expansion and contraction of a projected disc to simulate motion in the looming axis caused an increase and decrease in luminous flux. Therefore, neurons responding to *L* axis motion could have been responding to luminance cues instead of, or in addition to, motion information. The luminance of the disc was not adjusted to maintain constant luminous flux during *L* axis animation because this would have changed the contrast of the disc edge. The luminance test determined the responses of object-sensitive units to luminance changes. The disc used in *L* axis motion animation had a luminance of 51 cd/m^2^; changes in its diameter caused a luminous change of 2.4 lumens. Luminance tests started with a 51 cd/m^2^, 5 cm disc which was maintained at that luminance for a 1 second pre-stimulus interval. The disc then dropped to 1.9 cd/m^2^ (the background value) and increased to 273.6 cd/m^2^ over a 1 second time period, causing a luminous change of 11.2 lumens. The disc then returned to its original luminance value (51 cd/m^2^). This stimulus was presented for at least three trials (with 1 second inter- and post-stimulus intervals).

### Frequency test

The frequency test stimulus was a projected 5 cm diameter disc oscillating at an amplitude of 0.5 cm and frequencies ranging from 0–6 Hz. This test was specifically designed to test response across the range of oscillatory frequencies that hawkmoths are able to track, not necessarily to explore the entire frequency response range of descending visually sensitive neurons. The test range is indeed behaviorally relevant: *M. sexta's* flower tracking ability declines sharply at 3 Hz (Sprayberry and Daniel 2007) and the diurnal hawkmoth *Macroglossum stellatarum* has difficulty tracking frequencies higher than 5 Hz (Farina et al. 1994). This test presented motion in each axis individually, *i.e.* the *H*-axis frequency test oscillated the position of a disc from left to right, the *V*-axis test oscillated position up and down, and the *L* axis test oscillated the size of the disc (expansion and contraction). This experiment used the same presentation paradigm as the direction test. The disc was held stationary for 1 second pre-, inter-, and post-stimulus intervals surrounding 1 second motion-animation periods. For all Vision Egg experiments, and for Psychophysics Toolbox experiments up through 3 Hz, frequency increased in increments of 0.5 Hz. However, because frequency of object oscillation was dependent on frame rate for the Psychophysics Toolbox experiments, presented frequencies higher than 3 Hz were 3.33, 3.75, 4.29, 5, and 6 Hz.

**Figure 2.**
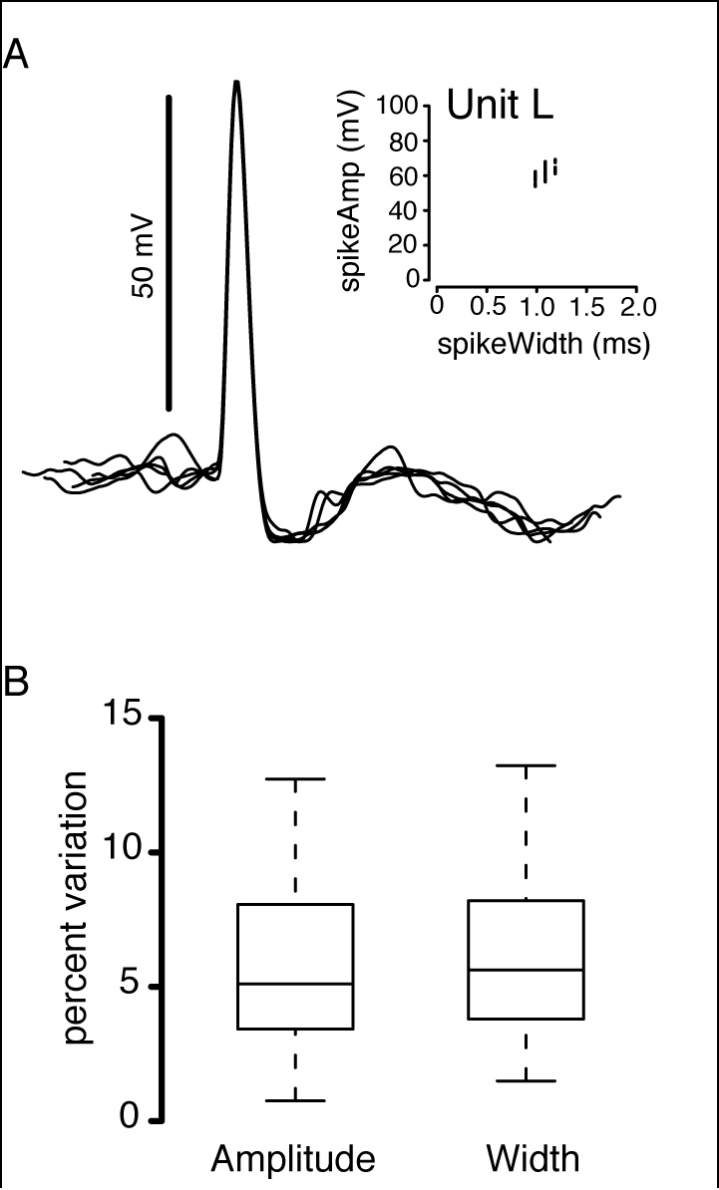
Only single unit recordings were analyzed in this manuscript. Recordings were defined as single unit if they displayed less than 15% variation in spike amplitude and width. (a) Overlaid traces of 5 spikes from unit L, which showed 3.34% variation in spike height and 4.14% variation in spike width, (b) A box plot showing the mean, standard error, and range of variation in spike amplitude and width of all included units.

### Analysis

All cells included in this analysis showed depolarized spikes (Figures 1, 2), indicating that the recording electrode had likely penetrated the cell. However, there is no anatomical data to confirm intracellular recording, and the baseline shifts indicating cell penetration were not visible for recordings made on the extracellular amplifier. Spike waveforms were analyzed to confirm that recordings were indeed from single units; cell recordings needed to exhibit less than a 15% variation in both spike height and width to be included in this manuscript (Figure 2). Percent variation was computed as the ratio of the standard deviation to the median for both parameters. Additionally, this manuscript only considers those units that responded significantly to at least one direction of object motion presented in the direction test (ANOVA, Tukey's HSD, 95% CI).

### Latency analysis

The duration of movement in one direction of motion for a 6 Hz oscillating stimulus (*i.e.* a half cycle) was 83 ms. Latencies of optic lobe neurons can be 50 ms or more depending on luminance (Theobald 2004), and likely longer for descending neurons. Therefore, a reliable estimate of latency was necessary to accurately measure spiking responses to oscillating stimuli. The cross-correlation was calculated between the velocity pattern of the direction-test experiment and each unit's spiking response (measured as summed spikes for three trials in 1 ms bins). The latency was measured as the time lag associated with the maximum correlation value. The time window used to sample spike rates for all experiments was phase-shifted by the latency value for that individual unit.

### Direction selectivity

To examine how selective a unit was for a given direction of motion a specificity index was calculated for responses to the direction test, where the index (*I*) is equal to the response to an individual direction of motion divided by the sum of all responses (R):

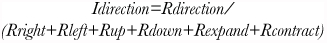

A value of 1 indicates that unit is only responsive to that direction of motion, whereas a value of 0.16 indicates that unit responds to all directions equally. This index was calculated for each unit's response to all six directions of motion presented in the direction test.

### Sensitivity vectors

To gauge the 3D distribution of direction sensitivities exhibited by recorded cells, 3D sensitivity vectors were calculated by creating a single sensitivity value for each axis of motion (*H, V,* and *L*). The normalized response to leftward motion was subtracted from that for rightward motion to yield a horizontal axis sensitivity value (*SH*)*.* To calculate vertical sensitivity (*SV*), the normalized response to upward motion was subtracted from that for downward motion, and for looming sensitivity (*SL*) the normalized response to an expanding disc was subtracted from that for a contracting disc:

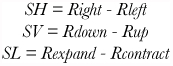

Each 3D direction sensitivity vector originates at (0,0,0) and terminates at the (*SH, SV, SL*) coordinates calculated for that unit.

## Results

### Photoreceptor responses

**Figure 3.**
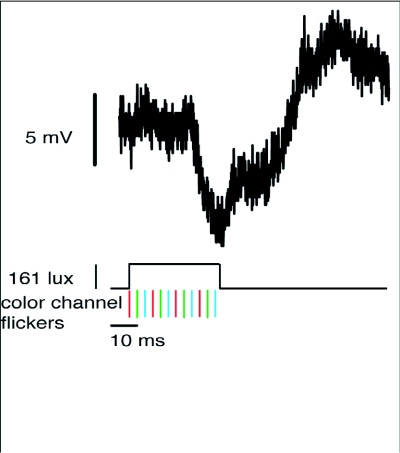
ERG trace from a *Manduca sexta* retina in response to a grey disc presented for 2 frames (33.33 ms). During a single frame (based on the 60 Hz information-update framerate), the projector goes through two cycles of flickering in each individual color channel (I frame = RGBRGB flickers). The average luminance is shown by the solid black trace under photoreceptor responses. The flicker of the individual color channels is shown by the colored hash marks beneath the luminance trace. The single ERG in response to the flash indicates the moth's retina did not resolve the 120 Hz flicker present in each color channel, nor did it resolve the overall 360 Hz flicker of the projector.

The DLP projector used for these experiments had a 120 Hz luminance flicker in each color channel, and an overall 50% luminance flicker of 360 Hz. ERGs were recorded from three *M. sexta* to test for a 120 Hz flicker artifact. If the retina was responding to the 120 Hz flicker present in any of the color channels, multiple ERGs would be evident in response to the presented 2—frame flash of light. However, as shown in Figure 3, the retina exhibited a single distinct ERG in response to the flash. While data from only one *M. sexta* is presented, the data from the remaining two animals were consistent with these results. This indicates *M. sexta* do not resolve either the 120 Hz flicker of individual color channels or the 360 Hz overall luminance flicker.

### Responses to the direction test

**Figure 4.**
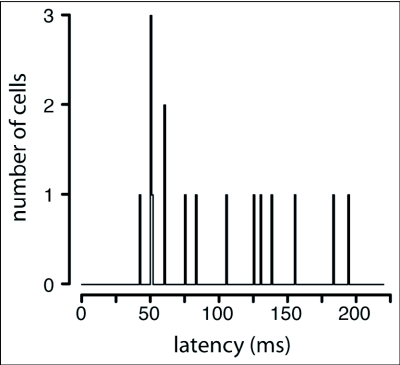
A frequency histogram showing the response-latency distribution of the 16 object sensitive descending neurons (OSDNs) of *Manduca sexta*. The majority of units had latencies under 100 ms, with peaks in the 50 ms range.

All units were tested for their response to six different directions of object motion with a 5 cm disc that moved left—right (*H* axis), up—down (*V* axis), and approach—recede (*L* axis). Over 100 moths were recorded from during the course of these experiments, yielding approximately 50 visually-sensitive cells in 23 different animals. Of those 50 recordings, 26 responded significantly to object motion (ANOVA, *p* < 0.05). Three of those 26 failed the statistical requirements for single-unit recordings and seven were discarded because of technical errors, leaving 16 object motion-sensitive cells in 12 different animals. The latency values for these units ranged from 42 to 200 ms, with over half the units exhibiting latencies in the 40–90 ms range (Figure 4). Those units having longer latencies were not associated with any other response properties (direction, frequency, or sinusoidal grating tuning). Object-sensitive descending neurons (OSDNs) show relatively low baseline spike rates. Of the 16 units, 2 had zero baseline activity. Excluding a single outlier (whose baseline activity was 52 Hz, and had responses up to 95 Hz), baseline spike rates for OSDNs ranged between 1 and 12 Hz, and responses to the direction test ranged as high as 54 Hz.

**Figure 5.**
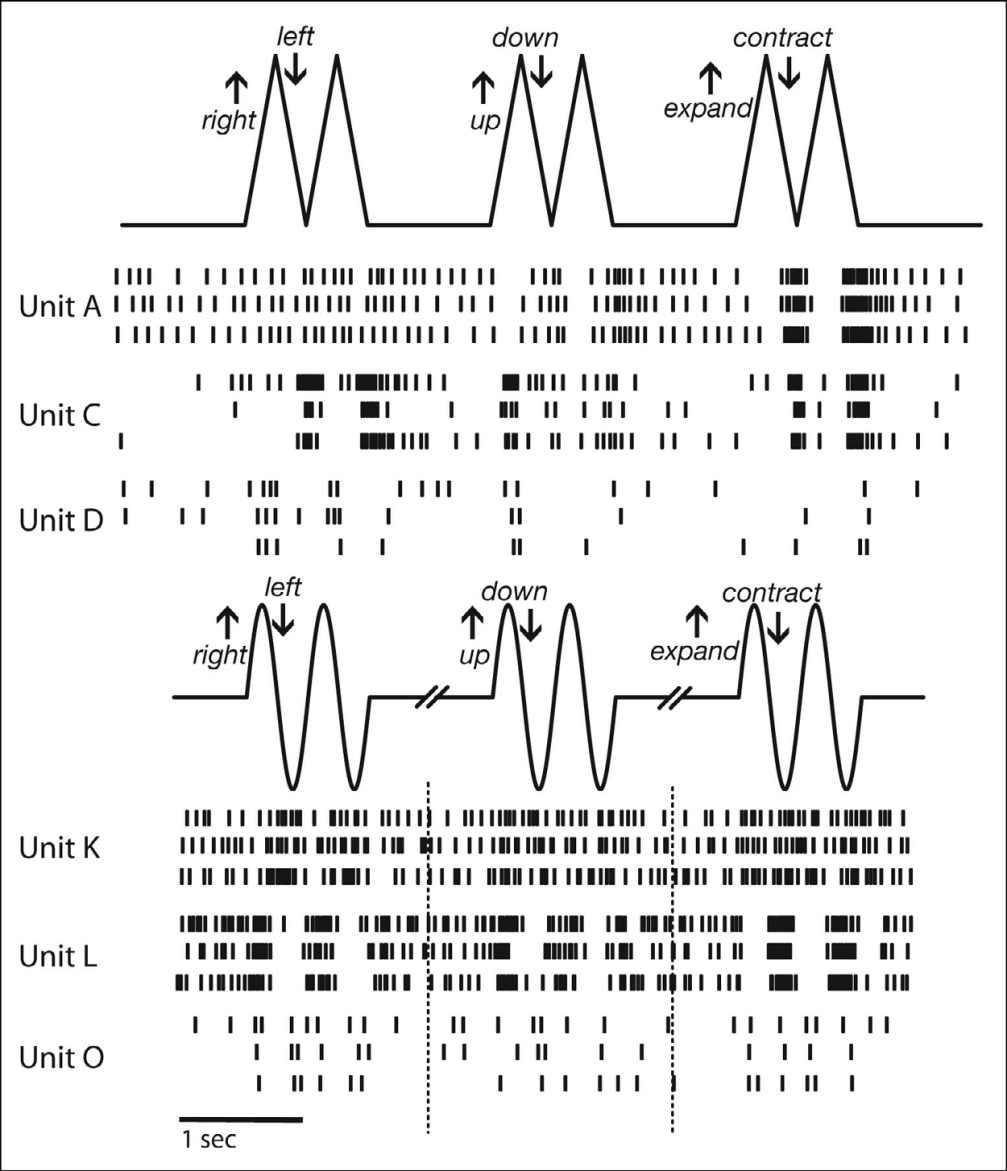
A raster plot of the response of six different *Manduca sexta* units to the direction test. The top trace shows object position during the direction test as coded in the Matlab Psychophysics Toolbox experiments. Raster trains of response for three different units (three trials for each) are shown beneath the position trace. The second solid trace shows object position as coded in the Vision Egg experiments. Again, raster trains of response for three different units (three trials for each) are shown beneath the position trace. For both types of direction test (saw-tooth and sine-wave position profiles), OSDNs showed relatively consistent spike timing across trials.

**Figure 6.**
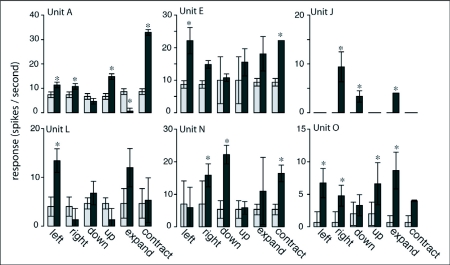
Sample responses of *Manduca sexta* object sensitive descending neurons (OSDNs) to the direction test, where the dark grey bars are spiking responses to the labeled direction of motion and the light grey bars represent the corresponding baseline spike rate. OSDNs typically responded to multiple directions of motion. However, no consistent patterns of directional sensitivity were found. Responses to a given direction of motion that were significantly different from baseline are denoted with an asterisk (ANOVA, Tukey's HSD, p<0.05).

**Figure 7.**
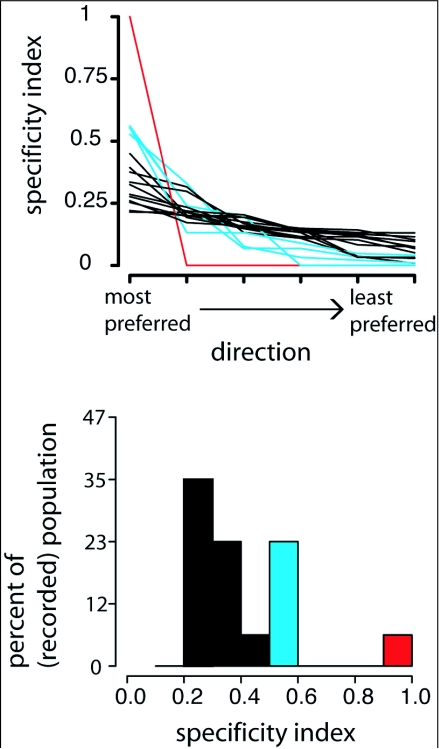
Direction selectivity of units in *Manduca sexta* can be assessed by calculating a selectivity index (*l*) for each unit's responses to the direction test. *I* values near I indicate a highly selective unit, while *I* values closer to 0 indicate sensitivity to multiple directions of motion. (A) *I* values of each unit were calculated and sorted according to their selectivity so that each line represents the relationship of *I* values for responses to different directions by a single unit. Highly selective units (red lines) have steep slopes, moderately selective units (blue lines) have moderate slopes, and weakly selective units (black lines) have very shallow slopes. (B) Constructing a histogram of the highest *I* value for each unit, the OSDN population sorts itself into three rough classes of selectivity: high (red), moderate (blue) and weak (black)

As shown by a raster plot for six different units, spiking responses to the direction test were relatively consistent from trial to trial (Figure 5). Although inhibitory responses appear rare, occurring significantly only in unit A (Tukey's HSD, 95% CI: 4.611.3), statistically insignificant but easily visible suppression of response to certain directions of object motion did occur in several other units (as seen in Figure 5). The dominant response characteristic of OSDNs was sensitivity of individual units to multiple directions of motion, without any repeating patterns of preferred directions between units (Figure 6). To more closely examine direction selectivity of OSDNs, a selectivity index *I* was calculated for each unit's response to all six directions of motion. An *I* value close to 1 indicates that unit is highly selective for that direction, and an *I* value close to 0 indicates that unit is nonselective for that direction. The *I* values for each unit are sorted from high to low and plotted in Figure 7A. Each line illustrates the relationship between *I* values for different directions within the same unit, with a shallow slope indicating that unit responds to multiple directions of motion. Constructing a histogram of *I* values for each unit's most preferred direction, we can see that the OSDN population sorted itself into three rough classes (Figure 7B): 1. highly direction selective (1 unit, 6 % of the population); 2. moderately direction selective (4 units, 25% of the population); 3. weakly direction selective (11 units, 69 % of the population).

**Figure 8.**
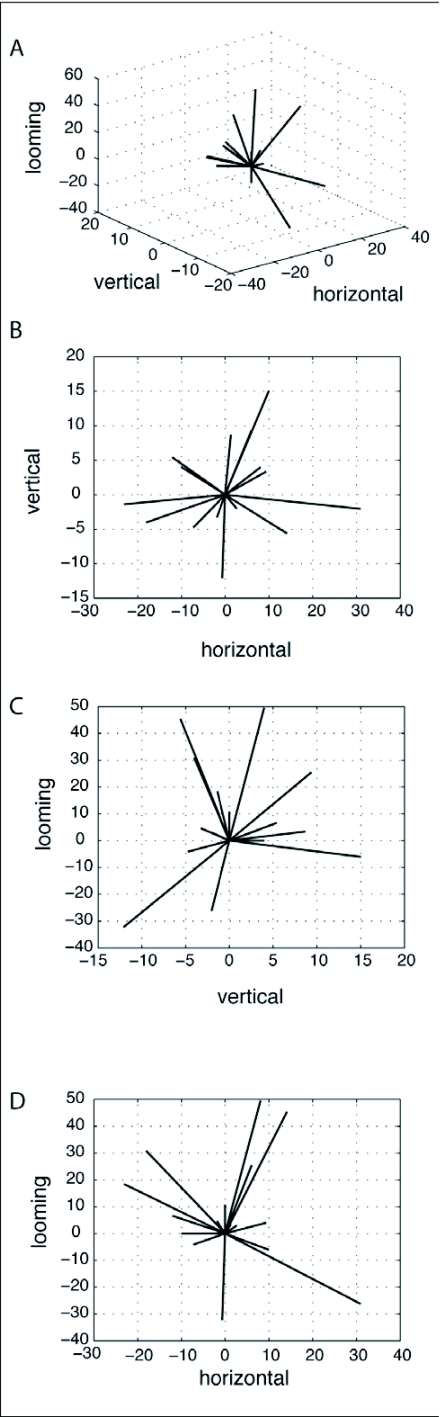
OSDNs showed a broad spatial distribution of direction sensitivities in *Manduca sexta*. A 3D directionalsensitivity vector for each unit was obtained by assigning a single sensitivity value for each axis of motion: horizontal axis = *Rright* - *Rleft,* vertical axis = *Rdown* - *Rup,* and looming axis = *Rexpand* - *Rcontract.* The distribution of these sensitivity vectors is shown in a 3D (A), as well as the three projected 2D plots: the vertical axis plotted against horizontal (B), looming against vertical (C) and looming against horizontal (D).

The tendency to respond to multiple directions of motion does not exclude the possibility of having distinct classes of directional tuning. When represented in a two dimensional format, such as bar graphs (Figure 6), OSDNs did not demonstrate clear classes of directional tuning. Given that units were tested with three dimensions of motion, it was possible that representing OSDNs' directional sensitivities in 3D space would reveal classes of directional tuning. To check for this, a 3D directional-sensitivity vector was calculated for each unit. The 3D directional-sensitivity vectors of OSDNs exhibited a broad spatial distribution, with no clear grouping into classes of directional tuning (Figure 8). The purpose of these sensitivity vectors was not to determine a single preferred direction vector for a unit but to instead represent the spatial distribution of direction sensitivities.

These sensitivity values can also be used to roughly examine the distribution of directional preference within each axis of motion. For example, a positive *SH* value would indicate a preference for rightward motion. As shown in Table 1, there is an even distribution of directional preferences for the horizontal and vertical motion axes. Within the looming axis there are a greater number of cells that respond to approaching discs than receding discs. Many visual studies classify horizontal motion as ipsilateral and contracteral, as opposed to right and left. Whether right motion is ipisilateral or contralateral depends on which connective the OSDN resides in (Figure 1). While we see an even distribution of direction sensitivity on the horizontal axis when using the classification of right minus left response (Table 1), recalculating the H sensitivity value using ipsilateral minus contralateral response could reveal a different pattern. However, the recording preparation for these experiments made it difficult to determine which connective the electrode was recording from. Even if the electrode enters in one connective, it can easily slip into the adjacent connective given the geometry of the micromanipulator and projection screen arrangement. Therefore, units cannot be classified as responding to ipsi- or contra-lateral motion, only right and left. However, the primary purpose of this study is to elucidate what information is passed from the optic ganglia to the thoracic flight control system. To that end, the data currently indicate an even distribution of right—left sensitivities.

**Table 1.**
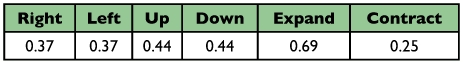
Ratios of units that exhibited a particular direction sensitivity based upon horizontal (SH), vertical (SV), and looming (SL) sensitivity values. Because SH was calculated as (response to rightward motion) - (response to leftward motion), a unit with a positive value is considered to be ‘right sensitive’. Likewise, units with negative values are considered to be ‘left sensitive’; and so on for SV and SL. Units with a sensitivity value less than +/- the standard deviation of baseline spike rate are considered to have a sensitivity value of 0, and are not included in the count for that axis.

### Responses to the luminance test

**Figure 9.**
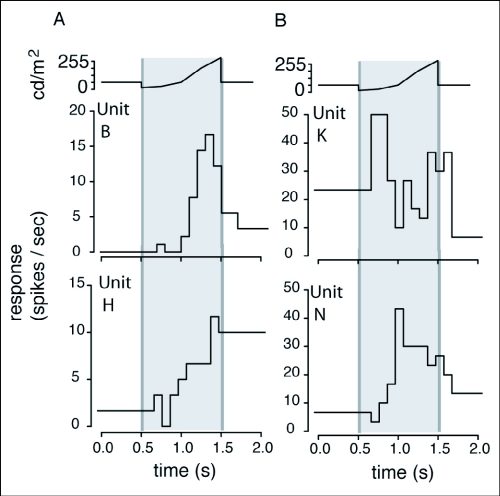
OSDNs exhibited two types of luminance response in *Manduca sexta*: (1) what would be predicted based upon their response to an expanding and contracting disc, and (2) the opposite of what would be predicted. The traces above the response plots indicate the luminance of the stimulus, which was a 5 cm disc centered on the moth's field of view. The response plots show spikes per second (calculated in 10 ms bins) plotted against time. (A) Two examples of OSDNs responding to a luminance test as would be predicted from their response to looming axis motion. Both units B and H responded to an expanding disc and increasing luminance. (B) Unit K responds to an expanding disc and decreasing luminance, while Unit N responds to a contracting disc and increasing luminance.

Animating motion in the *L* axis caused changes in luminance; an approaching/expanding disc increased total luminance, and a receding/contracting disc decreased luminance. Because luminance changes could be used by OSDNs to encode *L* axis motion, units were subjected to a luminance test in which a stationary disc drops to background luminance then increases to bright white over a 1 second time period. If indeed OSDNs consistently use luminance cues to encode *L* axis motion, units that respond to an approaching disc should also respond to increasing luminance. In fact OSDNs exhibited both the expected response based upon *L* axis motion preferences, and the opposite response. For example, unit B responded to an expanding disc and increasing luminance (Figure 9a). However, unit N responded to a contracting disc and increasing luminance (Figure 9b). Of the ten units subjected to the luminance test, four exhibited the predicted response and three had the opposite response. Two units could not be classified: one responded to both approaching and receding discs and decreasing luminance, the other responded to approaching discs but exhibited an ambiguous response to the luminance test. The last unit, unit I, did not respond to luminance changes.

### Frequency tuning of OSDNs

When recording time permitted, OSDNs were tested with varying frequencies (0 6 Hz) of object motion. The aim of these experiments was to explore frequency responses in a behaviorally relevant range.

**Figure 10.**
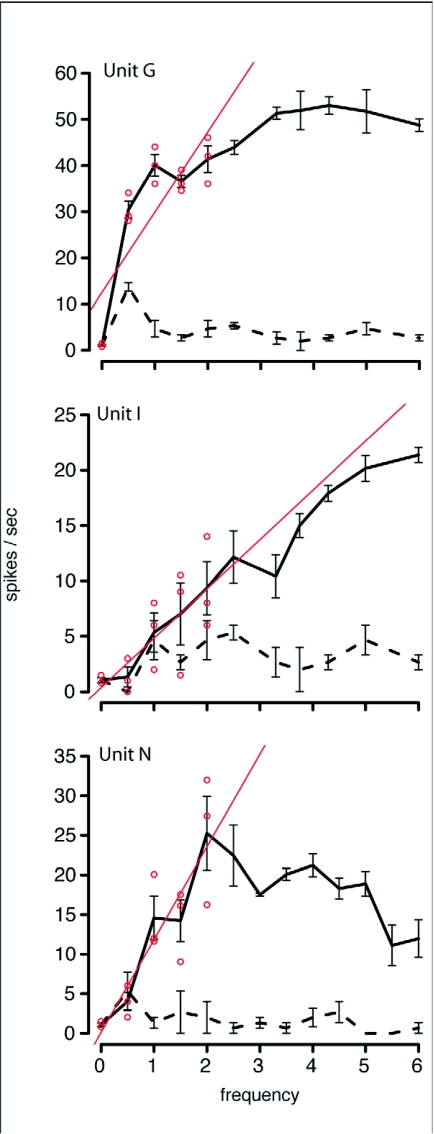
OSDNs showed broad tuning to varying frequencies of object motion in *Manduca sexta*. Unit I was an exception to this pattern, showing a monotonically increasing response to the frequency test. Spiking responses to the stimulus are shown as a solid line, while baseline spike rates are represented by the dashed line. The red line through each response curve represents the regression line that was fit to the 0–2 Hz response data. The red circles indicate the raw data points the regression line was fit to. The regression analysis indicates that for the range of frequencies that *M. sexta* can track (0–2 Hz), response increased with frequency (coefficients for this analysis can be found in Table 2).

Successful frequency response curves were obtained from 6 units in 5 animals. With one exception (unit I showed a monotonically increasing response to increasing frequency) OSDNs exhibited broad tuning over the tested range of frequencies (Figure 10). However, tracking ability in *M. sexta* falls off at frequencies higher than 2 Hz (Sprayberry and Daniel 2007). Interestingly, a linear regression fit to the frequency response data in this behaviorally relevant range (0–2 Hz) returns a significantly positive slope for 5 of the 6 units tested (Table 2). Therefore, given moving flower stimuli that *M. sexta* are capable of tracking, we see increasing response to increasing frequency. This response typically plateaus or gradually tapers at frequencies of 2–6 Hz.

**Table 2.**
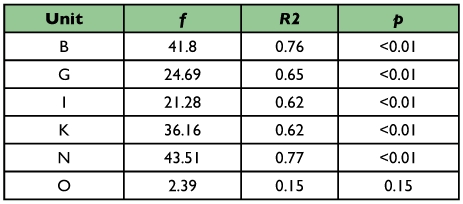
Statistical coefficients for linear regression on OSDNS response to the frequency test from 0 – 2 Hz.

## Discussion

Flower tracking is a robust visually-guided flight behavior in hawkmoths. In order to effectively track a flower, the hawkmoth's nervous system needs to transmit information about that flower's motion to the musculature. Directional information, i.e. where to go, is critical for orchestrating this behavior. Secondarily, information about how far and how fast to move in a particular direction could increase the precision of tracking behavior. To examine what visual information is indeed available to the flight musculature, individual object-sensitive descending neurons (OSDNs) in the cervical connectives were tested with different directions and frequencies of oscillatory object motion.

Responses of optic-lobe neurons show distinct tuning to direction of motion, with a clear preferred direction (eliciting a strong response) and null direction (inhibition in response to the opposite direction of the preferred direction). Prior studies on neurons arising in the optic ganglia of hawkmoths have revealed three broad classes of direction sensitivity (horizontal cells, vertical cells, and class 1 looming cells), with six subclasses (progressive, regressive, up-senstive, down-sensitive, approach-sensitive, and recedingsensitive) (Wicklein and Varju 1999; Wicklein and Strausfeld 2000). Classification of horizontal and vertical cells was performed using wide-field gratings, but Wicklein and Strausfeld (2000)'s investigation into looming detectors used looming discs, rotating spirals, and wide-field gratings (animating motion in the horizontal and vertical axes) as visual stimuli. In addition to the class 1 cells, which responded solely to approaching or receding discs, they described a class of centrifugal neurons that arise in the posterior slope and transmit information to the optic ganglia. These class 2 neurons responded to rotating spirals and wide-field moving gratings in addition to object motion in the looming axis. They exhibited a preferred and null direction for each axis of motion, i.e. excited by an approaching disc and inhibited by a receding one, excited by rightward motion and inhibited by leftward, and excited by upward motion and inhibited by downward. Direction tuning properties of OSDNs are markedly different from described optic lobe neurons: they respond to multiple directions of motion with varying response strengths (Figure 6) without repeatable patterns of direction sensitivity. Within an individual axis of motion, OSDNS exhibit a range of behaviors, with some units showing a preferred and null direction, some responding to both directions and some showing no response to motion within that axis. Given these response properties, OSDNs do not appear to be maintaining the basic classes of direction tuning described in the optic lobe. OSDNs are likely integrating responses from multiple cell classes, (potentially the ‘Class 1’ cells described by Wicklein and Strausfeld (2000) and the analogues of the horizontal and vertical wide-field cells described in the hawkmoth *Macroglossum stellatarum* by Wicklein and Varju (1999)). While neuroanatomical studies are needed to further investigate this, Rind (1983a) has previously described a visuallysensitive descending neuron in *M. sexta* whose input branches are located in the protocerebrum where they could synapse with optic lobe outputs.

Although an individual OSDN's response to direction of object motion does not provide unambiguous encoding, the broad spatial distribution of direction sensitivities, (Figure 8) indicates that accurate direction information may be available at the population level. There is precedent for population control of steering behavior in insects: neural control of turning behavior in cockroaches is based on the response of multiple neurons (Levi and Camhi 2000). An individual neuron's response has a proportional effect on turning, as opposed to an all-or-none effect. The neuromuscular control of flower tracking could be organized in a similar manner, so that the hawkmoth's path is determined by responses of the entire OSDN population. This question is a topic for future studies.

When tested with varying frequencies of object motion, OSDNs do not exhibit strong selectivity. These broad frequency response curves would seem to indicate that OSDNs are not robustly encoding frequency of flower motion (Figure 10). However, *M. sexta*'s tracking ability sharply decreases above 2 Hz (Sprayberry and Daniel 2007). When looking at frequency response in this behaviorally relevant range, OSDNs exhibit a monotonically increasing response. Therefore it's likely that frequency information is indeed being passed to flight control centers.

When comparing results of this study with prior behavioral work, it appears that flower tracking is not limited by the visual system. For instance, flower tracking ability is not uniform across directions. *M. sexta* track flowers moving in the looming axis worse than those moving in the horizontal and vertical axes, exhibiting a decline in ability at frequencies higher than 1 Hz. However, based upon OSDN responses to the direction test, accurate information about all three axes of motion is being transmitted to thoracic flight control centers. Additionally, while frequency information may be less accurate above 2 HZ, OSDNs still exhibit robust responses to those higher frequencies. It is more likely that tracking ability in *M. sexta* is mechanically and/or aerodynamically limited.

## Abbreviations

**OSDN**: object-sensitive descending neuron
**ERG**: electroretinogram
H: horizontal
V: vertical
L: looming - toward and away
